# Editorial and Miscellaneous

**Published:** 1862-11

**Authors:** 


### EDITORIAL AND MISCELLANEOUS.

*Twentieth Session of Rush Medical College.*—The Introductory exercises of this Institution took place according to the Announcement on Wednesday evening, Oct. 1st. The lower lecture room of the College building was filled to overflowing by students and friends of the School. Each chair was represented on the platform, Dr. Brainard presiding, and the Rev. D. C. Locke, Rector of Grace Church, acting as Chaplain.

Owing to the unexpected detention of Prof. R. L. Rea, then acting as fleet surgeon on the Mississippi, the Annual Introductory Address expected from him was given by Dr. Allen. As it is published, by request of the class, we forbear any comments upon it in this place. We barely suggest the editorial regret that Prof. Rea had not occupied the hour and our pages—for whatever that gentleman undertakes always savors strongly of the sound and right—even when he lashes a mulish Brigadier.

An unexpectedly large class is in attendance, and, without any disparagement to previous classes, we must be permitted to say it is one of which any College might well be proud. Many of them are preparing for the position of surgeons and assistants in the army. This, under the circumstances, is peculiarly gratifying. It is well known that the present "Examining Board" of applicants for surgeoncies in the army is made up from the faculty of what is called the "Med. Dep. of Lind University," and it is notorious that the interests of the soldiery and of the commonwealth have throughout their entire term of service been held immediately subordinate to those of the *secession school.* The idea has been assiduously inculcated that to pass the Examining Board it is incumbent on students first to make friends with the individuals of that Board by attending the skeleton college, which they are vainly attempting to prevent from the usual fate of mortality. A

few weak medical infants have succumbed to this "pressure," but, we are happy to state, but a small fraction of the number in attendance upon "Old Rush." The secession dodge has again failed.

We congratulate the alumni of the College in that, at least, *one hundred and fifty* are now in attendance upon the annual lectures.

---

*The College Cliniques.*—The semi-weekly Cliniques of the Rush Med. Coll., attended by Profs. Brainard and Allen, are largely supported by patients. In no way has the advantage of this great central city of the Northwest for clinical instruction been so remarkably exemplified as in this immediately practical part of the instruction in the College. It suffices to say that the number in attendance has been so great as often to render it impossible to bring all the cases before the class in the hours devoted to that purpose. Patients, both surgical and medical are attended every day at the College, and public clinics take place semi-weekly. The City Hospital, under the immediate supervision of Profs. Freer and Ingals, also affords a good field for practical observation and study. We can confidently assure the profession that this great desideratum in medical teaching is rapidly taking a place in Chicago which will be surpassed by scarcely any other city in the country.

---

*Return of Dr. Holmes.*—We are pleased to meet again upon the street the face of our genial friend and correspondent, Dr. E. S. Holmes. (For a glimpse of the particulars, see p. 467 of this volume.) Another of the Dr.'s letters appears in the present number, and others will be given from time to time.

---

*Prescriptions.*—Great care is requisite in writing prescriptions that they may be appropriately adapted to the age, etc., of the patient. The following left to the family "to be suggested" to the attending physician for a patient, *fifteen months old* only, seems eminently Malthusian: ℞. Hyd. Cum Creta ℈j, Pulv. Opii gr. j, Pulv. Camph. gr. iv., Pulv. Doveri gr. x. M. In chart, No. iv. S. Give one every three hours. The attending physician failed to see the propriety of giving the infant a half grain of opium and a gr. of camphor, at all events quite so often.——In a case of Puerperal Convulsions, recently, a medical man of large power of secession discourse, prescribed: ℞ Calomel gr. x, Morph. Sulph. gr. ij. M. In chart. vj. divid. S. One every hour. So great was his confidence in the combination that he refused any further attendance or treatment—insisting that if that did not cure nothing would. From the result—it seems "nothing would." ——In another case the same gentleman having diagnosed tumor of the posterior wall of the uterus—a state of things he remarked, which he had "never before seen in so young a woman"—prescribed *Liquor Potassæ*, confidently assuring the patient that he "could cure her in three or four years." A few weeks after, he was sent for in great haste, and found that a miscarriage had just taken place—thus establishing a new influence of Liquor Potassæ, well worth bearing in mind. The objurgations of the patient and family, it is reported, were particularly enlivening to the bystanders. The tumor has wholly disappeared. The family physician had previously diagnosed pregnancy.——We saw recently a prescription consisting essentially of Morph., Hyoscy, and Syr. Scillæ Comp., amounting to a pint, to be given in teaspoonful doses, which both the "Dr." and the druggist (probably in silent partnership) asserted could not be put up in less amount. The large cost of the mixture announced by the druggist staggered the patient, who incontinently retreated, and afterwards our friends Buck & Rayner put up one-fourth of the amount at a fair sum. The profession and all honorable druggists owe it to themselves to discountenance such imposition. The one given is but a sample from scores which have been given to us. We shall recur to the subject again.

---

*Woman before the Examining Board.*—A woman lately applied for examination to enter the Med. Dep. of the army to the Examining Board. Strangely enough they decline the honor. Our friend, Dr. R—g—s, says that he was fairly nonplussed the other night, in passing the catheter to relieve the bladder of a six foot masculine, whilst a buxom lass of twenty summers held the candle—but we suppose necessity knows no law.

*Vesico-Vaginal Fistula.*—Prof. DeLaskie Miller has within the last few weeks operated on three cases of this distressing lesion, in each case with a successful result. He has promised us a full report in an early number of the JOURNAL.

---

*Wheeler & Wilson*—the advertisement of whose incomparable Sewing Machine appears on the cover of the JOURNAL—have removed their establishment to new and magnificent quarters, at 106 Lake street. It is well worth the while of all visitors of the city to look in at their superb store. We question whether in beauty, adaptation, or a certain *je ne sais quoi* of refined elegance, its superior can be found on the continent. It is a capital exponent of the *recherche* taste and culture of the gentlemanly superintendent, Geo. R. Chittenden, Esq., who originated both the general design and the minor appointments of the edifice.

The W. & W. Machine took the highest premium at the recent World's Fair in London—an honor richly deserved.

---

*Death of Dr. E. S. Cooper, of San Francisco.*—The telegraph brings the melancholy intelligence of the death of this gentleman, well known to our readers as a former resident of this State, and as a voluminous contributor to surgical literature. No particulars of his illness have as yet been received.

---

CHLORIDE OF LIME is well known as one of the very best remedies for wounds and sores, where there is a want of healthy action or a tendency to gangrene; also for deep burns, etc. It is generally used by mixing with cream or the fixed oils in the proportion of about one to ten of oil; but its volatile nature has prevented its use in any preparation ready to be applied. This difficulty is now obviated in the following preparation:

Take any of the resins of proper consistence, or Rosin and a volatile oil; Spts. Turpentine or Petroleum in such proportion as to make a wax that may be worked with the fingers; mix thoroughly with from five to ten per cent of Chloride of Lime, previously moistened by one-third of its weight of water. The whole enters into chemical combination, forming a nice adhesive paste, soothing, stimulating and affording perfect protection to the parts. It is prepared for the trade under the name of "Magic Healer." A preparation in market called "Rapid Healing Balsam" is made from the same formula. L. J. GILDERSLEEVE.

